# An Insight into Amorphous Shear Band in Magnetorheological Solid by Atomic Force Microscope

**DOI:** 10.3390/ma14164384

**Published:** 2021-08-05

**Authors:** Mohd Aidy Faizal Johari, Asmawan Mohd Sarman, Saiful Amri Mazlan, Ubaidillah U, Nur Azmah Nordin, Siti Aishah Abdul Aziz, Norhasnidawani Johari, Nurhazimah Nazmi, Shahir Mohd Yusuf

**Affiliations:** 1 Engineering Materials & Structures (eMast) ikhoza, Malaysia-Japan International Institute of Technology (MJIIT), Universiti Teknologi Malaysia, Kuala Lumpur 54100, Malaysia; mohdaidyfaizal@graduate.utm.my (M.A.F.J.); amri.kl@utm.my (S.A.M.); nurazmah.nordin@utm.my (N.A.N.); aishah118@gmail.com (S.A.A.A.); norhasnidawani@utm.my (N.J.); nurhazimah@utm.my (N.N.); shahiryasin@utm.my (S.M.Y.); 2 Civil Engineering Department, Faculty of Engineering, Universiti Malaysia Sabah, Kota Kinabalu 88400, Sabah, Malaysia; 3 Mechanical Engineering Department, Faculty of Engineering, Universitas Sebelas Maret, J1. Ir. Sutami 36A, Ketingan, Surakarta 57126, Central Java, Indonesia; ubaidillah_ft@staff.uns.ac.id

**Keywords:** atomic force microscopy, magnetorheological solid, microplasticity, shear band, stress relaxation

## Abstract

Micro mechanism consideration is critical for gaining a thorough understanding of amorphous shear band behavior in magnetorheological (MR) solids, particularly those with viscoelastic matrices. Heretofore, the characteristics of shear bands in terms of formation, physical evolution, and response to stress distribution at the localized region have gone largely unnoticed and unexplored. Notwithstanding these limitations, atomic force microscopy (AFM) has been used to explore the nature of shear band deformation in MR materials during stress relaxation. Stress relaxation at a constant low strain of 0.01% and an oscillatory shear of defined test duration played a major role in the creation of the shear band. In this analysis, the localized area of the study defined shear bands as varying in size and dominantly deformed in the matrix with no evidence of inhibition by embedded carbonyl iron particles (CIPs). The association between the shear band and the adjacent zone was further studied using in-phase imaging of AFM tapping mode and demonstrated the presence of localized affected zone around the shear band. Taken together, the results provide important insights into the proposed shear band deformation zone (SBDZ). This study sheds a contemporary light on the contentious issue of amorphous shear band deformation behavior and makes several contributions to the current literature.

## 1. Introduction

The development of magnetorheological elastomer (MRE) materials and their advancement over the years of breakthrough in materials science has had a significant influence on the material revolution. Categorized as intelligent and receptive solid materials, MRE has properties that can be substantially modified by external magnetic stimuli. However, MRE is still not as common as other viscoelastic materials despite its smart characteristics, primarily due to substantial limitations in the current experimental and theoretical research on these materials [1]. It is only recently that evidence of a qualitative link between the theoretical mixture model and magnetorheological fluids (MRF) experiments has been obtained [2]. MRE consists of a mixture of two materials of different kinds. It consists of magnetizable particles, such as iron powders or carbonyl iron particles, which are immersed in an elastomeric material, and during the curing process, the material will transform into the desired shape [3]. The initial microstructural condition of the cured mixtures plays important role in determining the performance of the MRE. Microscopic-scale analysis has successfully observed this initial microstructure configuration and alignment of the particle and how it contributed to the enhancement of the field dependence of the mechanical properties of MRE when subjected to the applied force and magnetic field [4,5,6,7]. Moreover, the microscopic analysis also is the art and science of examining the mechanism of failed components to determine the cause of failure. It is one of the major steps in the process of post-failure analysis [8,9,10,11,12]. Therefore, few methods have been used to observe the microscopic analysis including optical microscopy. To date, optical microscopy was a popular method of identifying the microstructure of materials. However, more advanced and promising tools have recently been introduced for highly detailed microscopic images, including electron and scanning probe microscopy [13,14,15,16,17]. The use of microscopy analysis has been extensively used to explore in the study of post-failure rheological features of the MRE due to the precision of the measurement and the highest image resolution quality [18]. 

At the microscopic scale, forces in MRE are defined using micro stress and strain, and the distribution of stress applied during the test is the most commonly studied parameter for both elastic and plastic deformation of MRE. There has been an increasing number of publications [8,9,10,19] concentrating on a microscopic scale in MRE, with micro stresses ranging from one part of a molecular chain to the other. Additionally, it is very important to demonstrate the rheological behavior of MREs, particularly the time-dependent rheological nature of MRE and stress releases under constant strain, known as stress relaxation properties in detail microscopic analysis, in order to design a material that has good durability. Stress relaxation tests are, therefore, important to demonstrate the rheological viscoelastic behavior of MRE durability and to provide guidance on their application [20]. Previously, studies related to stress relaxation have attracted significant critical interest in natural rubber, modified rubber, nanocomposites, and amorphous solid [21,22,23,24,25,26,27]. However, in the stress relaxation investigation based on MRE, only a few have been published [20,28]. 

Generally, the molecular structure was in the strained condition for some finite duration during the stress relaxation process, resulting in some amount of strained microplastic [26]. Micro plasticity deformation then took place through a series of local reorganizations. However, due to their localization at a significantly low strain value, the region only deformed plastic in a very narrow area, resulting in the development of the shear band. Much attention has been paid to the shear bands phenomena, especially in metallic glasses, amorphous solids, and granular materials, because of their key feature, which controls the process of plastic deformation [8,9,11,26,29,30,31,32,33,34]. The susceptibility of shear band propagation in MRE limits the extent of exploitation of their use in the application of engineering and limits their scope of application to non-primary structural components. By decreasing the mechanical properties and limiting the component’s longevity, the shear band can precede failure. Therefore, several studies [8,9,10,11,19,29,31,35,36,37,38] have examined the formation of shear bands in solid amorphous materials. Analytically, Dasgupta et al. [9] demonstrated the mechanism for shear localization under shear stresses in metallic glasses, which was the appearance of the structured plastic flow of the amorphous solid into a shear band. The research was also successfully evaluated and demonstrated that the shear banding mechanism could only occur at stress values that surpass the yield stress. Meanwhile, another investigation by Cao et al. [35] on understanding the nature of plasticity has shown that the shear band that occurred in the granular system (container roughened by glued glass particles) consisted of significant plastic behaviors and consequences of the shear band regime. Evidence from the experimental analysis of Hamm et al. [31] on randomly packed granular medium with brass beads, identified the form of the shear bands in the early stages and the evolution of the shear bands was found to be discontinuous. At a longer duration, Shen et al.’s [11] study on ferromagnetic metallic glass found that the shear band affected zone consisted of a nanoscale shear band, a micrometer-scale severely deformed zone in the vicinity of the shear band, and tens of micrometers of extended strain gradient region. Surprisingly, no numerical, modeling, or experimental analyses have yet been conducted on the shear band mechanism and deformation for the MRE. 

The debate continues about the best strategies for the characterization of the shear band. To date, there has been little agreement on determining the precise nature of the shear band. The available literature revealed that no previous study has investigated the shear band formation under stress relaxation. This has made the shear band more exclusive and unique. In the literature on shear band deformation, the relative importance of morphological observation has been subject to considerable discussion. Few published studies [8,10,37,38] have attempted to describe the nature development of the shear bands and structural evolution utilizing microscopy instruments. A recent study by He et al. [8] has been carried out using transmission electron microscopy (TEM) to examine the local conditions in shear bands. Nevertheless, TEM has its downsides, regardless of the advantages. It is necessary to cut the sample thin enough but still able to withstand the analysis process, for electrons to pass through the sample. Thus, partial shear band spatial distribution information within the matrix can be obtained. However, this means that in a single TEM image there is minimal sensitivity to depth. On the other hand, a few studies [11,12,18,23,39,40,41] have proven the use of the Atomic Force Microscope (AFM) that offered additional capabilities and emulate advantages over other microscopy instruments. The study of Shen et al. [11] may have been the first to be recorded on the evaluation of the shear band using AFM. A comprehensive review by De Sousa et al. [12] summarized that AFM can, therefore, also evaluated the fundamental properties of the sample surfaces, along with elastic properties, and effectively distinguished between phases of the scanned region. Another advantage of AFM is that it offers access to material morphology without the need for rigorous sample preparation. However, no AFM application has been implemented to date in order to better understand the shear band phenomenon in MRE. Therefore, motivated by the distinctive characteristic of the shear band that has undergone stress relaxation, AFM is jointly used for evaluating the additional features of shear bands after deformation in MRE. Moreover, a unique finding of an exclusive shear band deformation zone (SBDZ) is thoroughly examined using AFM.

The aim of this study was to determine the shear band characteristics in a system caused by localized microplasticity. The deformation characteristic of the shear band within the elastic region of the material was the most obvious finding to emerge from the analysis. Since the effects of the shear band on the neighboring matrix were not well understood, AFM images may provide information that is hidden in a topographic image.

## 2. Materials and Methods

MRE with silicone-rubber-based and carbonyl iron particle (CIP) filler has been established for the stress relaxation investigation and topography of fault mechanism region evaluation. The sample was manufactured using the traditional method by means of the required cylindrical closed mold. At a controlled speed of 200 rpm, the soft CIP (d50 = 3.8–5.3 μm, CC grade, supplied by BASF, Ludwigshafen, Germany) was mechanically stirred into silicone rubber (NS625tds) supplied by Nippon Steel Co., Tokyo, Japan, at room temperature 25 °C. With the application of a 0.1 wt% curing agent, the ready mixture of the 30 wt% matrix and 70 wt% CIP was left to be cured. The curing method took 2 h to generate a sample for experiment purposes. Finally, a circular disc-shaped sample of MRE was cut out using a hollow hole punch tool from the original prepared MRE disc sheet to the desired diameter of 20 mm, with a nominal thickness of 1.2 mm for dynamic oscillatory shear testing. Most importantly, the highly sharp and sturdy punching process has no effect on the sample because the sample also has a soft and easy-to-cut physical characteristic. As a result, there were no chances of internal stresses affecting the sample as a consequence of the very low applied load during the punching process.

The characteristic of resilience was obtained as a measure of resistance to microstructural shear deformation under fluctuating stress. The stress, strain, normal force, frequency, and length of the test period parameters were preliminarily confirmed by experimental rheological behavior. The storage modulus was determined for the specified sample test geometry from the measured relationship of proven torsional oscillatory shear theories. Usually, stress relaxation has been defined in terms of stress deterioration over time under constant strain conditions. Thus, the recent advances in stress relaxation methods have facilitated the investigation of the behavior of the MRE. The MRE sample was tested under torsional shear mode and measured by an oscillation parallel plate rheometer (Physica MCR 302, Anton Paar Company, Graz, Austria). The rheometer was set to the desired test condition (temperature, force, and gap) and a rotary disc parallel plate (pp20 rod) was mounted before the investigation. Once the sample was placed on the stationary base mount of the rheometer, it was preloaded to prevent it slipping through the wall. The scope in this study, is the strain level of 0.01% was decided from our earliest rheological study on the sample. The value was achieved from the determination of linear viscoelastic (LVE) limit. Based on the rheological results of the previous investigation, the loss modulus has no significant impact on the behavior of the MRE in the LVE region. The consequence of the elasticity condition is that the storage capacity is dominant rather than the extremely low value of the loss modulus and the loss factor for the dissipation of energy. Throughout the test, the shear deformation was continuously set at 0.01%; this was highlighted as the pristine attempt at the stress relaxation test for MRE closest to its state of rest and follows Newton’s first law, regarded as a condition of equilibrium. A constant test frequency of 1 Hz was set to preferably replicate the actual working condition in the application and to take into account the shear velocity gradient affected during the test. To allow for a broader range of behavior observation, the time interval of each test condition was set at every 4000 cycles, and the total length of the test reached up to 115,000 cycles.

By means of tapping-mode AFM study, observation for morphological studies on the MRE sample was further investigated. Using a Nanosensor tapping-mode monolithic-silicon AFM probe-type single-beam cantilever supplied by BudgetSensors, Sofia, Bulgaria., this open-loop mode was worked on a NanoWizard 3, NanoOptics AFM (JPK Instruments, Berlin, Germany). The cantilever had a nominal length of 125 μm and a nominal force constant of 40 N/m and a resonance frequency of 300 kHz. The cantilever’s uncoated tip has a revolving shape with a height of 17 μm and a radius of less than 10 nm. The initial scan area was set at 100 μm, and the comprehensive evaluation of the sheared sample with a specific failure mechanism was developed at a scan area of approximately 20 μm. For the calculation and phase images, bundled analysis software was manipulated, while AFM images have been processed using the JPK Instruments data processing software (Version SPM-5.1.8). 

## 3. Results

**Figure 1:**
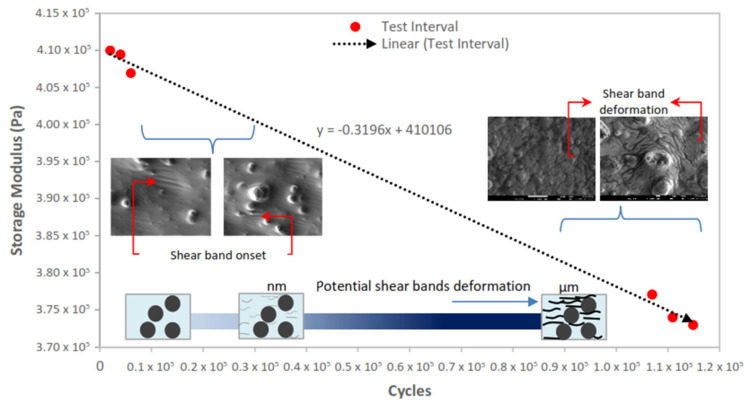
Storage modulus performance at the cycle-to-cycle fluctuation of oscillation shear stress for overall test cycles of stress relaxation. The supplemented linear correlation trendline led to the visual pattern spotting, nevertheless not completely representing the behavior. The left and right inserts display the FESEM image of shear band onset and deformation, respectively, at y=−0.3196x+410,106 transition. The lower insert indicates the potential size deformation of the shear band corresponding to the length of the test cycle.

The consistency of the MRE sample was first assessed by its ability to elastically store deformation energy through the characterization of the storage modulus. As shown in Figure 1, the continuous stress applied to identical constant strain has steadily diminished the proportionality activity of the MRE, and the covalent cross-linkages within matrix molecular structure have undermined the return-ability of the stretched chains.

As shown in the negative value of the slope variable in the linear equation correlation for the overall test cycles, the storage modulus value displays a decreasing pattern. These results were somewhat surprising considering the fact that energy storage capacity within the elastic region could still be impaired, even though the test was performed under a constant strain. The results showed that the ability of MRE to store deformation energy decreased marginally. There are several possible explanations for this result. The most important explanation is related to the relaxation of stress in the matrix’s amorphous molecular structure. On the molecular scale, stress relaxation could have occurred and been involved in the alteration of the molecular chain’s structure. In addition, stress relaxation phenomena in MRE have theoretically occurred through a number of mechanisms, including cross-link disengagement, elastic stretching, inelastic deformation, structural shift by phase transformation, structural rearrangement due to rupture, separation of microphases, microplasticity, and finally the nucleation of shear bands formation by localized strain. In this study, the onset of shear bands was observed as the earliest 2000-cycle test duration and consistently developed throughout every interval. 

The graph plotted in Figure 1 simultaneously indicates that the comparability evaluation of this activity corresponds to the durability of the early phase and the final interval range. For the start of the evaluation, a smaller interval is chosen to ensure that the onset of the shear bands is captured as early as possible. Choosing the broader interval during the initiation process of stress relaxation is risky and may miss the occurrence of shear band nucleation. An extended comprehensive test period for MRE stress relaxation evaluation could be introduced after the development of shear bands has become adequately consistent. In order to understand the complete cycle of MRE resilience to stress relaxation, while the shear bands have been consistently developed, the test was carried out continuously over a longer duration of the test cycle. The continuing concern within the LVE region of MRE is the impact of the persistent low strain at 0.01%. These findings indicated that the relationship of stress relaxation plays an important role in the MRE life cycle and revealed that the storage modulus decreased by approximately 10% over the overall total specified test duration. In the MRE stress relaxation analysis, this cycle scale was the longest ever recorded compared to the previous stress relaxation study [28]. 

**Figure 2:**
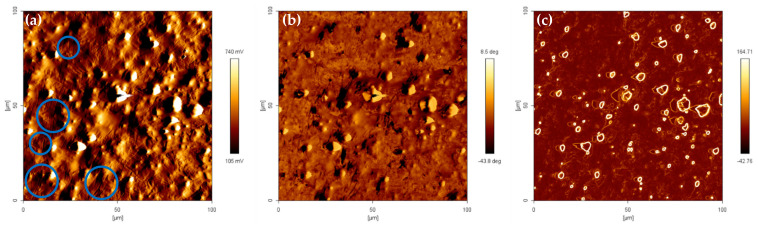
AFM images of over 100 µm scan area of oscillatory sheared MRE at 115,000 cycles obtained by tapping method, (**a**) amplitude channel, (**b**) phase channel, and (**c**) phase channel with edge detection.

The investigation by AFM in-phase images allowed us to be assured of the measure dissemination of micro-scale particles of the CIP within the elastomeric matrix. The AFM images of the sheared sample at 115,000 cycles are shown in Figure 2. After undergoing stress relaxation of the stated duration, the particles dispersed through the matrix can be seen protruding. The particle topography picture in Figure 2a was obtained with a drive amplitude of 0.075 V at a frequency of 263.916 kHz and 24.68 μm/s tip velocity on the scanned area of 100 µm × 100 μm. At this stage, the tiny line of shear band formation can be seen on the matrix within the area with lesser protruding particles on the left side of the image in Figure 2a. Further, the in-phase channel simultaneously reflects the modulus of the individual domain within the multi-domain (soft and hard) of MRE resulting from the stress relaxation shear durability test. The lower modulus of the softer domain presented in a darker color contrasting with the lighter color appearance of the harder domain. The phase image in Figure 2b was obtained at a phase shift angle of 165°. The variations throughout the phase image also indicate some kind of boundary between the soft and harder domain. As in Figure 2c, the image has been manipulated from the edge detection feature to reveal the edge of the particles. CIP appears to be evenly distributed over the matrix and varies in size. Utilizing the measurement tools in the software, it was found that the average of the smallest particle diameter was 1.5 μm, the medium diameter was 3.4 μm and the largest average particle diameter was 5.8 μm. The measurement was taken in the phase image at more than ten locations and validated to the specification of the CIP manufacturer.

**Figure 3:**
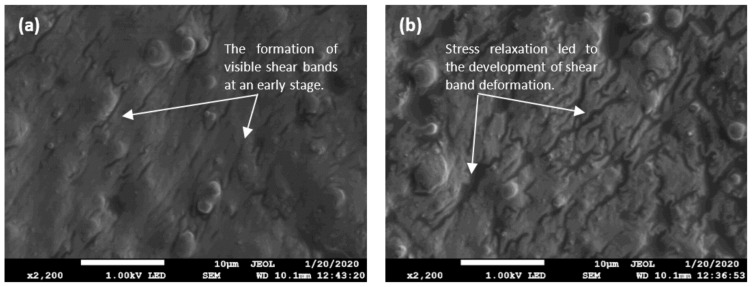
FESEM images of deformation micrographs at high magnification show a region with permanent deformation in the MRE matrix that remains localized in the confine shear band. (**a**) A micrograph of a part of an area indicating the presence of a small amorphous shear band onset. (**b**) A micrograph of a more visible shear band relief on the sample, consisting of several amorphous shear bands is sufficient evidence to summarize the mechanism’s characteristics after stress relaxation.

The present study was designed to determine the characteristic of the shear band in the system resulted from localized microplasticity. The most obvious finding to emerge from the analysis is that the deformation of the shear band within the elastic region of the material. This is also consistent with the observations through FESEM, as shown in Figure 3, which showed that shear band deformation involved with few stages and presented different physical characteristics during nucleation, Figure 3a, to a longer test duration, Figure 3b, where the formation of shear bands can be observed distinctly. This result, however, has not previously been described using AFM. AFM images may reveal information that is hidden in a topographic image, which may be attributed to the fact that the effects of the shear band on the neighboring matrix are not well understood. The elastic behavior of the matrix itself was originated from the cross-linking process in the amorphous molecular chain. This event happened in a very localized region. The continuous shear load has repeatedly smoothed the chains, causing the elastic limit in this region to be exceeded. Uneasily reconfigured with fewer cross-linkages and amorphous structure within the matrix domain originates from a micro-sized shear band.

**Figure 4:**
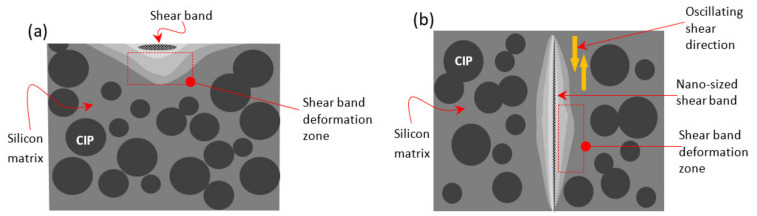
Schematic of the SBDZ developed from the localized microplasticity (**a**) cross-sectioned view and (**b**) top-view, as obtained from oscillated shearing of stress relaxation durability evaluation.

A consideration of the micro-mechanism is important for a deep understanding of the behavior of the shear band in the amorphous solid. This stage of research reveals the system or mechanism that influences the progression of the expanding shear band. It can, thus, be suggested that the ability to store deformation energy elastically through the storage modulus that was identified and measured from the experiment could be related to the shear band mechanism. It is possible to hypothesize that these conditions are likely to occur in the shear band deformation zone (SBDZ), as illustrated in Figure 4. In this zone, there is the formation of the sheared surface of the main shear band, micro-plastic dissipation due to micro-plastic zone formation in the matrix around the shear band edges, translation of the micro-plastic zone, and the secondary shear band that may develop parallel to the main shear band. Based on the schematic Figure 4a, the CIP embodied in the silicon matrix in the closest region to the shear band has produced secondary interaction. This interaction developed within the CIP surface and the localized enclosed matrix region provides a stronger bonding than just the matrix deformation zone itself. As a result, CIP has restricted itself from any movement or dislocation. Distributed micro-stress at continuous shear was then concentrated in a less effective localized area of SBDZ. Hypothetically, the SBDZ consists of variant cohesion regions. These variants can refer to the cohesive band surrounding the permanently deformed shear band. Oscillated shear in the direction, as shown in Figure 4b, has created stronger cohesive strength in the zone and drastically increased the shear-band width. Coherent intensity at higher contrast SBDZ repeats the process indefinitely, and the shear band thickness eventually depends heavily on the degree of initial inadequacy [42]. The SBDZ’s lightest contrast zone is then replaced by an increase in shear band thickness.

**Figure 5:**
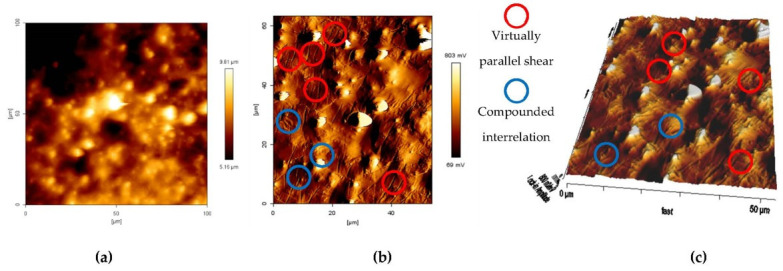
AFM topographic images of the shear band deformation in the MRE matrix (**a**) initial state of tapping-mode topographic image, (**b**) parallel and compounded interrelation shear bands, and (**c**) Three-dimensional topographic image of the shear band deformation.

Areas with several virtually parallel shear bands are created by micro-plastic deformation. The micro-plastic flow in the SBDZ and permanent micro-plastic formation of the inner portion at localized molecular chains are dependent on the state of the molecular structure and the crosslinked ability. The molecular structure at a similar elastic limit would have a similar pattern of formation of the shear bands parallel to each other. However, in the localized region with different elastic limit, will promote compounded interrelation of shear bands. Both conditions can be observed in the AFM topographic image in Figure 5. The scanned area images (100 µm × 100 µm) have a resolution of 512 × 512 pixels at a line rate of 0.157 Hz. The other potential cause for this difference was the stress relaxation mechanism, which has softened the molecular chain over a wide range of time. Unlike the process of increasing strain, stress relaxation is a very slow process of modifying the atomic arrangement. Shear plasticity deformation at this condition was believed to occur through a series of local reorganizations with a smooth and consistent operation. The materials have provided enough time to react to this very slow process. As the position of the strain deformed plastically at a very slow rate and only in a very narrow area, non-homogeneous deformation was, therefore, observed. Consequently, the general characteristic of the matrix, which is viscoelastic, limits the formation of the microplastic area.

**Figure 6:**
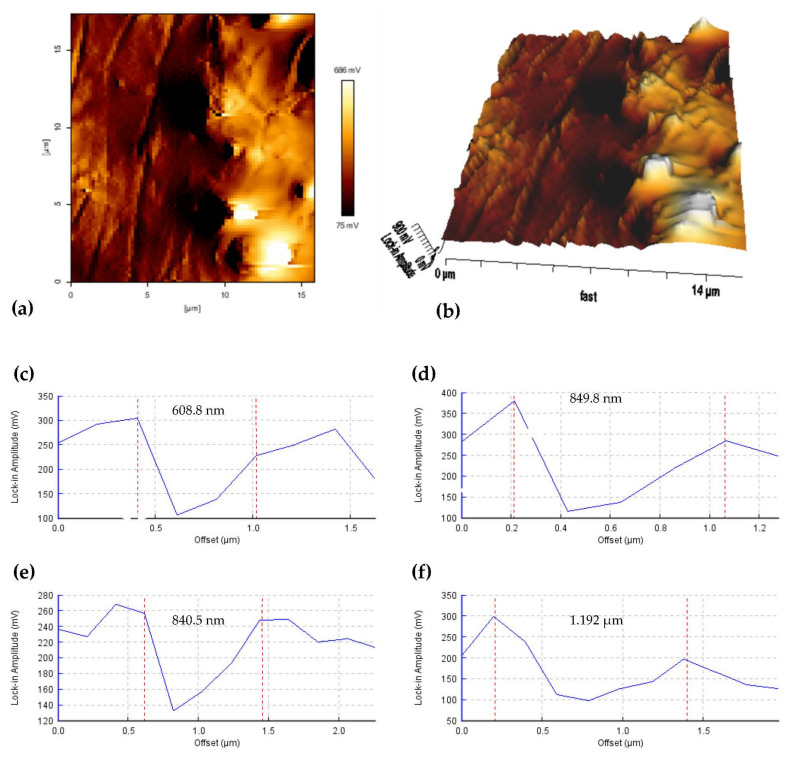
AFM amplitude topography image of the shear band deformation in the MRE matrix (**a**) nearly parallel and compounded interrelation shear bands; (**b**) three-dimensional topographic image of the shear band deformation. The red double-headed arrow represents the microphase separation. The bottom lock-in amplitude cross-sectioned graphs show the spacing thickness of the deformed shear band (**c**) the thinnest shear band of 608.8 nm (**d**) regular thickness value of 849.8 nm; (**e**) regular thickness value of 840.5 nm and (**f**) the thickest shear band of 1.192 µm.

A detailed observation of the shear band expresses itself in an outer region belonging to the elastic matrix domain, which is, thus, elastically strained as the stress in that domain falls below the elastic limit. Splitting the softening chains and tolerating harder elastic matrix domains resulted in microphase separation of elastic and microplasticity deformation. The microphase is representative of the micro-sized domain of the matrix, whereas the shear band falls within this phase separation. The separated spacing was measured, as shown in Figure 6, utilizing the AFM cross-sectioned image-measuring features. The deformed shear bands were measured with thicknesses ranging from 600 nm to less than 1.2 µm, which the smaller range identified populations within the lower-amplitude scale area.

**Figure 7:**
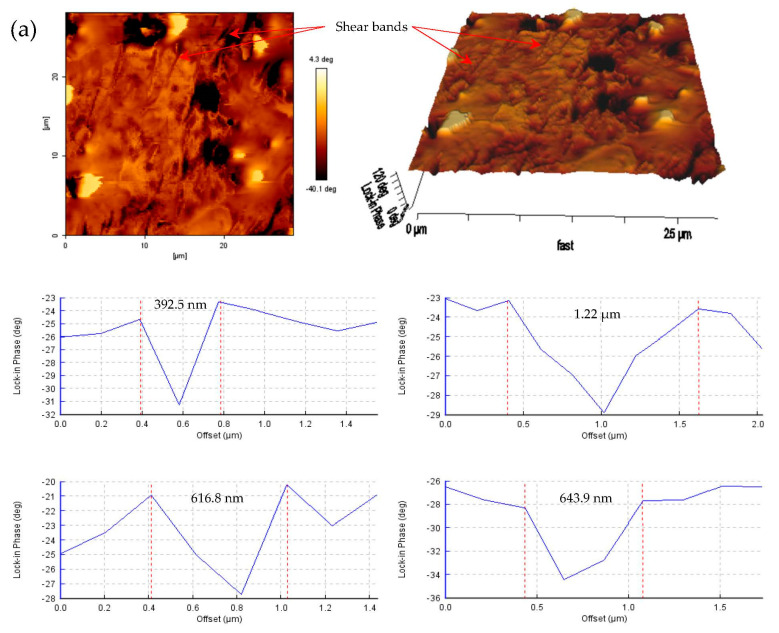

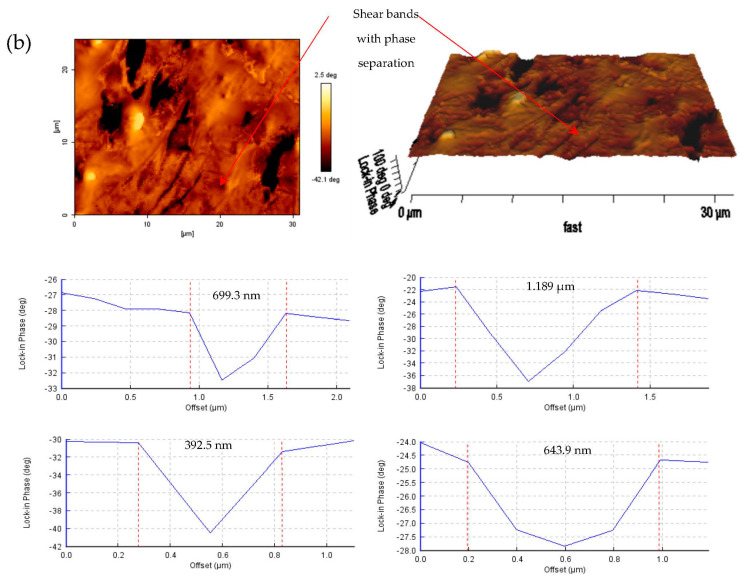
AFM taping-mode images of two- and three-dimensional in-phase topography of the shear band deformation zone. (**a**) Shear bands; (**b**) shear bands with a thicker phase separation.

In addition to measuring the drive amplitude, the in-phase images were mapped out to able to systematically be presenting the individual qualitative domain modulus. Figure 7 shows the phase images of approximately 30 µm × 30 µm scanned area of the shear bands deformation. The images have lower pixels and resolution for a smaller scanned area (30 µm× 30 µm). The average value at a similar line rate (0.157 Hz) was an approximately 160 × 160-pixel image. The phase images are presented in both two- and three-dimensional views to provide images with different viewpoints for better accurate characterization. Phase-imaging AFM allows us to differentiate and identify the hard and soft domains of the scanned area. For instance, Figure 7a,b display shear bands of nano- to micrometer thickness, and each shear band is designated with a soft domain, whereas the nearby matrix in the SBDZ appeared to be a harder domain. The spacings between shear bands were approximately similar. This signifies that the lighter color is implied by a higher modulus domain of the matrix, whereas the lower modulus domain of the shear bands appears darker. The mechanism in the SBDZ was then confirmed by a thorough observation of the shear band phase image, suggesting a decreasing contrast in the shear profile on each band.

**Figure 8:**
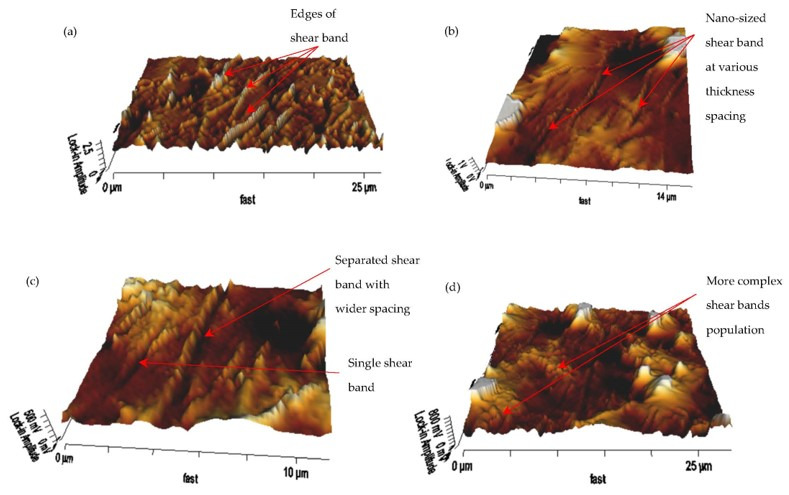
Three-dimensional AFM amplitude micrograph of the shear band deformation in the MRE matrix (**a**) edges detection, (**b**) nearly parallel shear band, (**c**) wider spacing of domain separation, and (**d**) complex shear band interaction.

Figure 8 provides a more detailed view of the unique and exceptional configuration of the shear band under stress relaxation. As illustrated in Figure 8a, one of the peculiar characteristics of the shear band can be determined by the edges formed on the nano-scale phase separation during this stress relaxation process. During the test where the fluctuating stress has broken the chain along with the cross-linked soft-dominated domain, the edges were formed. The nano-sized and various thickness spacing within neighboring bands, as shown in Figure 8b, were other distinct characteristics, due to stress relaxation. The strain was constant and optimally sheared throughout the test; however, in this region, the localization of strain was not persistent and contributed to the uncertainty of the plastic strain process, thus promoting a similar level of uncertainty in the shear band shape. The localization of the strain has become apparent by end of the test with the sighting of a thicker size shear band, as shown in Figure 8c. Nevertheless, due to the heterogeneity of localized stiffness of the cross-linked molecular structure of the matrix, few isolated shear bands with a small thickness can be observed within the region. Meanwhile, shear band deformation was not solely observed followed the maximum localized shear stress, but was affected by the maximum normal stress over the shear yielding or shear band formation. Therefore, as shown in Figure 8d, a more complex shear band population was disclosed by the AFM morphological observation.

## 4. Conclusions

This study set out to gain a better understanding of shear band deformation in an MRE amorphous matrix that had undergone stress relaxation. Thus, this study investigated the nature of the shear band by employing AFM as a novel method of measuring the features of the deformed shear band and the surrounding region. Under stress relaxation, the process of shear banding in the MRE matrix is primarily formed in the matrix, with no evidence of CIP inside the microphase shear band separation. AFM cross-sectioned features were used to measure shear bands ranging in thickness from 300 nm to less than 1.2 µm. The majority of shear bands are nearly parallel, with only a few having complex interrelationships, which may be due to the effect of maximum normal stress during the stress relaxation process. The evidence has demonstrated that the AFM in-phase images indicate the individual qualitative domain modulus within the shear band deformation region. Particularly, the insights gained contribute towards the enhancement of fundamental knowledge on the shear band in a number of ways and serves as a foundation for the proposed SBDZ mechanism. One of the study’s key strengths was the use of AFM in the morphological analysis of the single shear band characteristics and the deformation zone formed by stress relaxation. The method used offers valuable insights into the nano-scale shear band deformation region at an effectively three-dimensional view. The proposed novel mechanism is useful for the development of a framework in order to explore the complex relationship between the evolution of the MRE shear band and durability performance under stress relaxation. In the current study, MRE used was a solid and strain was applied purely in the elastic region, so the model only included the Hookean elastic spring model; the Maxwell model as the sample was subjected to stress relaxation. The physical-mathematical modeling of MRE will be considered as a possible next step in our investigation of this phenomenon and addition of new knowledge to this field.

## Data Availability

The raw/processed data required to reproduce these findings cannot be shared at this time as the data also form part of an ongoing study. In future, however, the raw data required to reproduce these findings will be available from the corresponding authors.
